# Neuromyelitis optica spectrum disorders with non opticospinal manifestations as initial symptoms: a long-term observational study

**DOI:** 10.1186/s12883-021-02059-1

**Published:** 2021-01-25

**Authors:** Rui Li, Danli Lu, Hao Li, Yuge Wang, Yaqing Shu, Yanyu Chang, Xiaobo Sun, Zhengqi Lu, Wei Qiu, Zhi Yang

**Affiliations:** 1 Department of Neurology, The Third Affiliated Hospital of Sun Yat-sen University, Guangzhou, China; 2 Department of Neurology, Maoming People’s Hospital, 101 Weimin Road, Maonan District, Maoming, Guangdong China; 3 Multiple Sclerosis Center, Department of Neurology, The Third Affiliated Hospital of Sun Yat-Sen University, No. 600 Tianhe Road, Guangzhou, Guangdong Province 510630 China

**Keywords:** Neuromyelitis optica spectrum disorders, Vomiting, Area postrema, Clinical outcomes, AQP4 antibody titers

## Abstract

**Background:**

Early stage neuromyelitis optica spectrum disorders (NMOSD) with non-opticospinal manifestations as initial symptoms are easily misdiagnosed; however, data on the full symptom profile are limited. Moreover, the clinical characteristics and long-term outcomes of these patients remain unknown. We sought to analyze the clinical characteristics, imaging features, and long-term outcomes of NMOSD with non-opticospinal manifestations as initial symptoms.

**Methods:**

We retrospectively included relevant patients from our center. Clinical, demographic, magnetic resonance imaging, treatment, and outcome data were compared according to the non-opticospinal vs. opticospinal initial symptoms.

**Results:**

We identified 43 (9.13 %) patients with non-opticospinal initial symptoms among 471 patients with NMOSD. Of these, 88.37 % developed optic neuritis/myelitis during an average follow-up period of 6.33 years. All the non-opticospinal symptoms were brain/brainstem symptoms. Most of the symptoms and associated brain lesions were reversible. These patients had a younger onset age (*P* < 0.001), lower serum aquaporin-4 (AQP4) antibody titers (*P* = 0.030), and a lower Expanded Disability Status Scale (EDSS) score at onset (*P* < 0.001) and follow-up (*P* = 0.041) than NMOSD patients with opticospinal initial symptoms. In addition, EDSS scores reached 3.0 (indicating moderate disability) later than in patients with opticospinal initial symptoms (*P* = 0.028).

**Conclusions:**

Patients with NMOSD with non-opticospinal initial symptoms have a younger onset age, lower serum AQP4 antibody titers, and better clinical outcomes.

## Background

Neuromyelitis optica (NMO) was first described by Devic and Gault over a century ago as a monophasic disorder characterized by bilateral optic neuritis (ON) and myelitis (MY) [1, 2]. The discovery of highly specific anti-aquaporin-4 (AQP4) antibodies established NMO as a distinct disease [3], which requires the presence of ON and MY for diagnosis [4]. However, subsequently, more restricted or more extensive central nervous system (CNS) involvement in NMO has been recognized, and the term NMO spectrum disorders (NMOSD) was proposed to encompass the entire clinical spectrum in the international consensus diagnostic criteria in 2015 [5]. The new criteria define a unifying diagnosis of NMOSD, which requires at least 1 of 6 core clinical characteristics in patients who are seropositive for AQP4 antibodies. The core clinical characteristics include ON, MY, area postrema syndrome, acute brainstem syndrome, diencephalic syndrome, and symptomatic cerebral syndrome with typical brain lesions. The new criteria extend the clinical scope of NMOSD beyond ON and MY and thus demand a high index of clinical suspicion in patients who present with non-opticospinal CNS manifestations. The frequency of non-opticospinal manifestations is far lower than that of opticospinal manifestations. A clinical analysis of the largest international cohort of patients with AQP4-seropositive NMOSD revealed that 84 % of cases presented with myelitis and 63 % with ON, while 15 % developed area postrema syndrome, 17 % developed brainstem syndrome, and 3 % developed diencephalic involvement during the disease course [6]. NMOSD with non-opticospinal manifestations as initial symptoms (NOSIS) are easily misdiagnosed in the early stage of the disease, and data on the full profile of NOSIS in NMOSD are limited. Moreover, the clinical characteristics and long-term clinical outcomes of patients with NMOSD with NOSIS (NMOSD-NOSIS) remain unknown.

Here, we describe the clinical characteristics and long-term clinical outcomes of patients with NMOSD-NOSIS. This study may provide insight into the pathogenesis and facilitate early recognition of NMOSD.

## Methods

### Case selection and identification

Patients admitted to the Department of Neurology of The Third Affiliated Hospital of Sun Yat-Sen University from January 2013 to June 2019 were included if they fulfilled the Wingerchuk NMO diagnostic criteria; patients who met the Wingerchuk 2006 criteria were reconfirmed based on the 2015 report [4, 7]. In addition, all participants had to have at least one follow-up visit at our center more than 1 year after the onset of symptoms. Patients with an uncertain AQP4-immunoglobulin G (IgG) status, disease duration < 12 months, insufficient information, or a history of other CNS diseases were excluded. All the patients with serum AQP4-IgG negativity fulfilled the criteria for negative AQP4-IgG NMOSD and their brain MRI lesions were not compatible with MS lesions, fulfilling MAGNIMS criteria [8]. Patients included in the study were divided into two groups: NMOSD-NOSIS and NMOSD with opticospinal manifestations as initial symptoms (NMOSD-OSIS). Fulfillment of the inclusion and exclusion criteria was confirmed retrospectively by a review of medical records by two neurologists specialized in demyelinating diseases (Yuge Wang and Wei Qiu).

### Clinical data

The patients’ medical records were reviewed retrospectively, and the following data were retrieved: age at onset, sex, annual relapse rate (ARR), coexisting autoimmune diseases, Expanded Disability Status Scale (EDSS) at onset and at the most recent visit, clinical manifestations, laboratory and MRI findings, and therapy including the use of azathioprine, mycophenolate mofetil, or rituximab. A relapse was defined as a new neurological deficit that lasted for > 24 hours and occurred > 30 days after the previous episode [9]. To analyze the frequencies of different relapses, we defined the following relapse phenotypes: isolated ON, isolated MY, simultaneous or sequential ON and MY occurring within 4 weeks, with or without brain/brainstem symptoms (ON + MY), isolated brain/brainstem presentations (brain/brainstem), and others (e.g., ON + brain/brainstem presentation, MY + brain/brainstem presentation).

### Laboratory tests

Serum AQP4 antibodies were tested using aquaporin 4-transfected cells from a commercial sampling kit (EUROIMMUN AG, Lübeck, Germany) according to the manufacturer’s instructions. Systemic disease diagnostic tests were routinely performed in most cases, including screening for anti-nuclear antibody, anti-Ro/SSA and anti-La/SSB antibodies, anti-double-stranded DNA antibody, anti-Smith antibody, anti-neutrophil cytoplasmic antibody, anti-U1RNP antibody, and rheumatoid factor.

### MRI scanning

MRI scans of the brain and spinal cord were performed on a GE 3.0 T or 1.5 T MRI scanner (General Electric, Milwaukee, WI), in the Radiology Department of the Third Affiliated Hospital of Sun Yat-Sen University. The imaging parameters for 3.0 T MR included: T1 with and without gadolinium enhancement (1,780/24.5 ms, repetition time (TR)/echo time (TE)), T2 (5,600/90 ms, TR/TE), and fluid attenuated inversion recovery (8,400/150 ms, TR/TE) sequences. The imaging parameters for 1.5 T MR were as follows: T1 with and without gadolinium enhancement (400/15.5 ms, TR/TE), T2 (2,500–3,500/100 ms, TR/TE), and fluid attenuated inversion recovery (8,800/120 ms, TR/TE) sequences.

### Statistical analysis

Statistical analyses were conducted using SPSS version 22.0 (SPSS, Chicago, IL, USA). Variables that conformed to the normal distribution were reported as means and standard deviations, and those that did not conform to the normal distribution were reported as medians and interquartile ranges (IQR). The two-tailed *t*-test, Mann-Whitney U test, χ^2^ test, and Fisher’s exact test were used to compare the NMOSD-NOSIS and NMOSD-OSIS groups, and the Wilcoxon two-sample test was used to compare pre- and post-treatment ARRs. The time to reach an EDSS score of 3.0 in the NMOSD-NOSIS and NMOSD-OSIS groups was analyzed using Kaplan-Meier curves. A *P-*value < 0.05 indicated statistical significance.

## Results

### Clinical characteristics and imaging features of patients with NMOSD-NOSIS

**Fig. 1:**
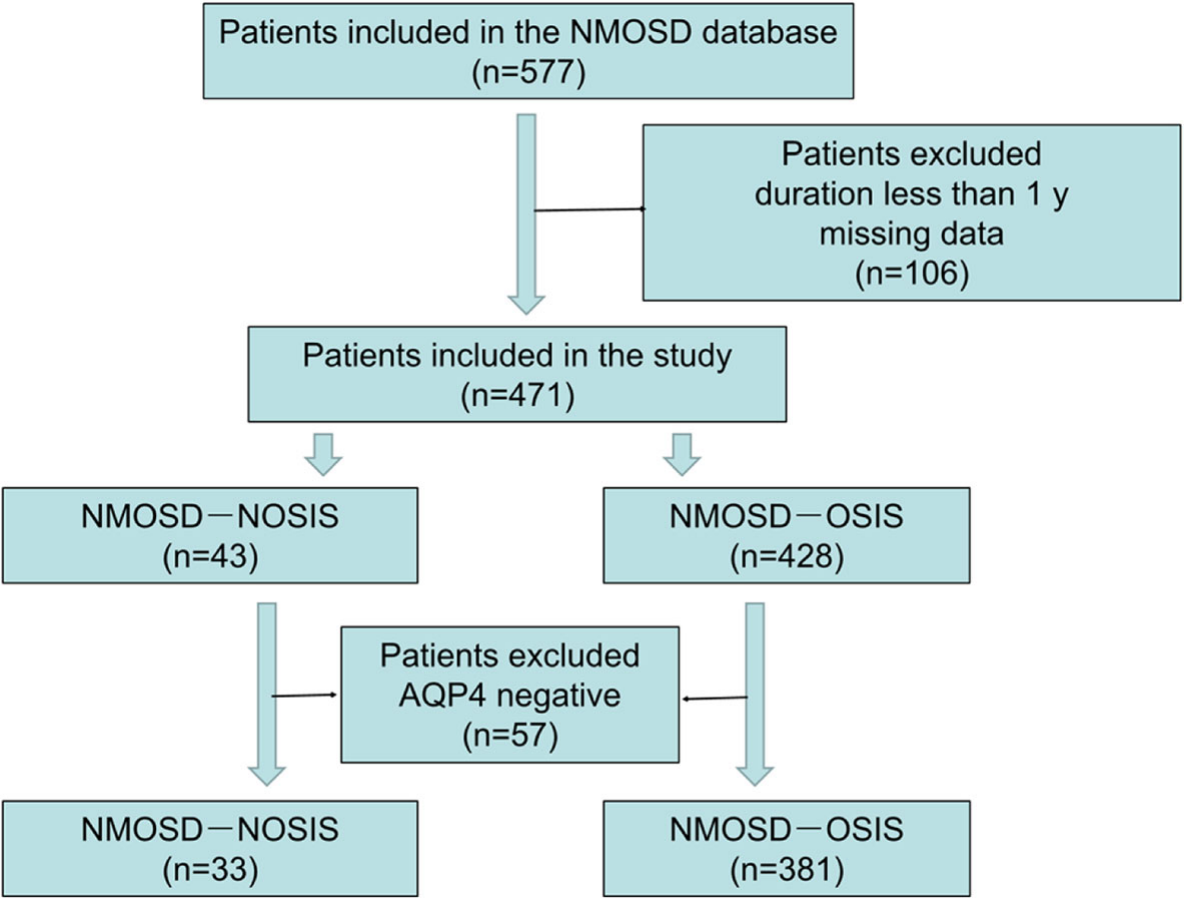
Study flow chart. NMOSD: neuromyelitis optica spectrum disorders; NMOSD-NOSIS: NMOSD with non-opticospinal manifestations as initial symptoms; NMOSD-OSIS: NMOSD with opticospinal manifestations as initial symptoms

**Fig. 2:**
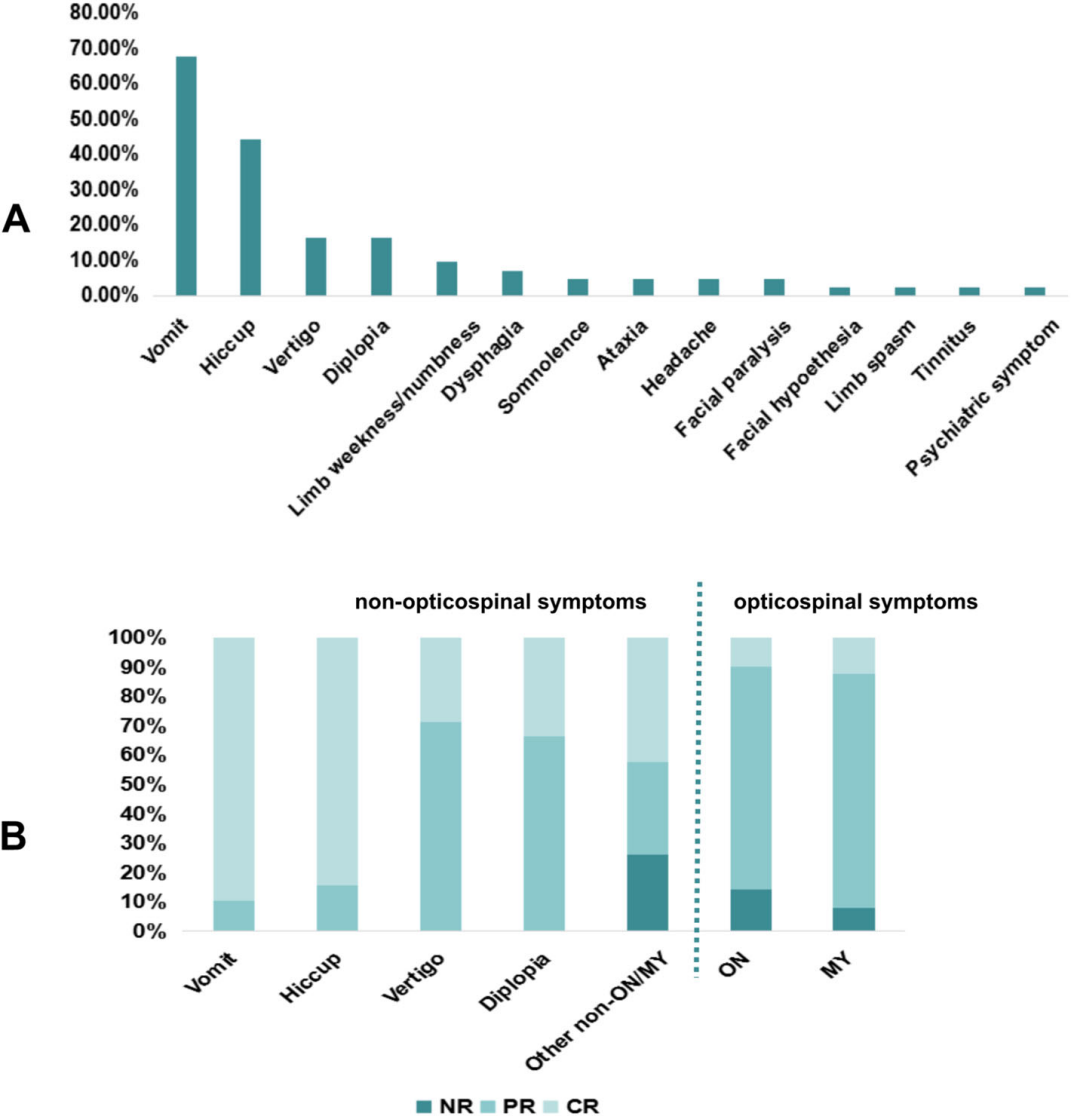
Analysis of non-opticospinal and opticospinal symptoms in NMOSD-NOSIS. **a**: The composition of non-opticospinal initial symptoms in NMOSD-NOSIS; **b**: The comparison of recovery between non-opticospinal initial symptoms and opticospinal symptoms in NMOSD-NOSIS. ON:optic neuritis; MY: myelitis; NR: no remission; PR: partial remission; CR: complete remission; NOSIS: non-opticospinal manifestation as initial symptoms; NMOSD-NOSIS: NMOSD with non-opticospinal manifestations as initial symptoms

We identified 43 (9.13 %) patients with NMOSD***-***NOSIS among 471 patients with NMOSD (Fig. 1). Table 1 summarizes the demographic and clinical features of the patients with NMOSD***-***NOSIS. Thirty-eight (88.37 %) of these patients developed ON/MY during the follow-up, while the other five patients showed only non-opticospinal manifestations during the follow-up period. The median time within which NMOSD-NOSIS patients developed ON/MY was 12 (IQR: 8–26) months. The median age at onset in the NMOSD-NOSIS cohort was 28 (IQR: 21–37) years. The median follow-up period at the last follow-up was 5 (IQR: 3–8) years. The serum AQP4-IgG positivity rate was 83.72 %. Serum Myelin Oligodendrocyte Glycoprotein-IgG was assayed in four out of seven patients with negative serum AQP4-IgG, and all were negative. The follow-up period of NMOSD-NOSIS patients who demonstrated only non-opticospinal manifestations was shorter than that of NMOSD-NOSIS patients who developed ON/MY (3.00 (IQR: 1.00–5.00) vs. 5.50 (IQR: 3.00–8.25), *P* = 0.042). There were no significant differences in other clinical manifestations and disabilities between the NMOSD-NOSIS patients who developed ON/MY and the NMOSD-NOSIS patients who showed only non-opticospinal manifestations during the follow-up period (Table 1). Among these NMOSD-NOSIS patients, 15 with vomiting or hiccups as initial symptoms first visited the Department of Gastroenterology, one with peripheral facial paralysis as the initial symptom was first diagnosed with facial neuritis, while two cases with dysphagia and quadriplegia were first diagnosed with Guillain-Barre Syndrome (GBS). Eighty-one non-opticospinal initial symptoms in these 43 NMOSD-NOSIS patients were analyzed. The most common non-opticospinal initial symptom was vomiting (67.44 %), followed by hiccups (44.19 %), vertigo (16.28 %), diplopia (16.28 %), limb weakness/numbness (9.30 %), dysphagia (6.98 %), somnolence (4.65 %), ataxia (4.65 %), headache (4.65 %), facial paralysis (4.65 %), facial hypoesthesia (2.33 %), limb spasm (2.33 %), tinnitus (2.33 %), and psychiatric symptoms (2.33 %) (Fig. 2a). Patients with somnolence did not fulfill the criteria for narcolepsy, which has been described as a symptom of NMOSD [7]. Fifty-seven non-opticospinal initial symptoms went untreated or were treated symptomatically, and 24 non-opticospinal initial symptoms were treated with high-dose intravenous corticosteroid therapy. Most of these initial symptoms resolved completely, even without steroid treatment in some cases. The total complete remission (CR) rate of these initial non-opticospinal symptoms was 66.67 %, including a vomiting CR rate of 89.66 % and a hiccup CR rate of 84.21 % (Fig. 2b).

**Table 1:** Comparison about the demographic and clinical features between NMOSD-NOSIS patients with different developments

		Total *n* = 43	NMOSD-NOSIS developing ON/MY *n* = 38	NMOSD-NOSIS restricted in non-ON/MY *n* = 5		*P* value
Age of onset, y, median(IQR)		28.00(21.00–37.00)	28.00(20.75–35.50)	26.00(22.50–42.00)		0.652
Female, n(%)		38(88.37)	33(86.84)	5(100)		0.904
AQP4-IgG+, n(%)		36 (83.72 )	32 (84.21 )	4 (80.00)		0.811
ARR, median(IQR)		0.75(0.50–1.27)	0.75(0.50–1.25)	0.67(0.48–3.00)		0.657
Coexisting autoimmunity, n(%)		6 (13.95)	5 (13.16)	1 (20.00)		0.592
CSF leukocyte cell count, n/ul, median(IQR)		0.30(0–4.00)	3.00(0–4.00)	1.00(0–14.00)		0.983
CSF protein, mg/L, mean(SD)		0.26 ± 0.13	0.26 ± 0.14	0.26 ± 0.11		0.980
EDSS at onset, median(IQR)		0(0–2.00)	0(0–2.00)	2.00(1.00–2.00)		0.356
EDSS at follow-up, median(IQR)		2.50(2.00–3.00)	2.75(1.75–3.00)	2.00(1.00–3.75)		0.622
Follow-up, y, median(IQR)		5.00(3.00–8.00)	5.50(3.00–8.25)	3.00(1.00–5.00)		0.042

*NMOSD* neuromyelitis optica spectrum disorders, *NMOSD-NOSIS* NMOSD with non-opticospinal manifestations as initial symptoms, *NMOSD-OSIS* NMOSD with opticospinal manifestations as initial symptoms, *IQR* Interquartile range, *ON *optic neurotis, *MY *myelitis, *AQP4* anti-aquaporin-4, *ARR *annual relapse rate, *CSF *cerebral spinal fluid, *EDSS *expanded disability status scale

To compare the difference in the recovery of non-opticospinal and opticospinal symptoms, we selected all the isolated ON relapses (*n* = 21) and MY relapses (*n* = 25) in these 43 NMOSD-NOSIS patients as controls. We found that the CR rate of non-opticospinal symptoms was higher than that of opticospinal symptoms (Fig. 2b).

**Fig. 3:**
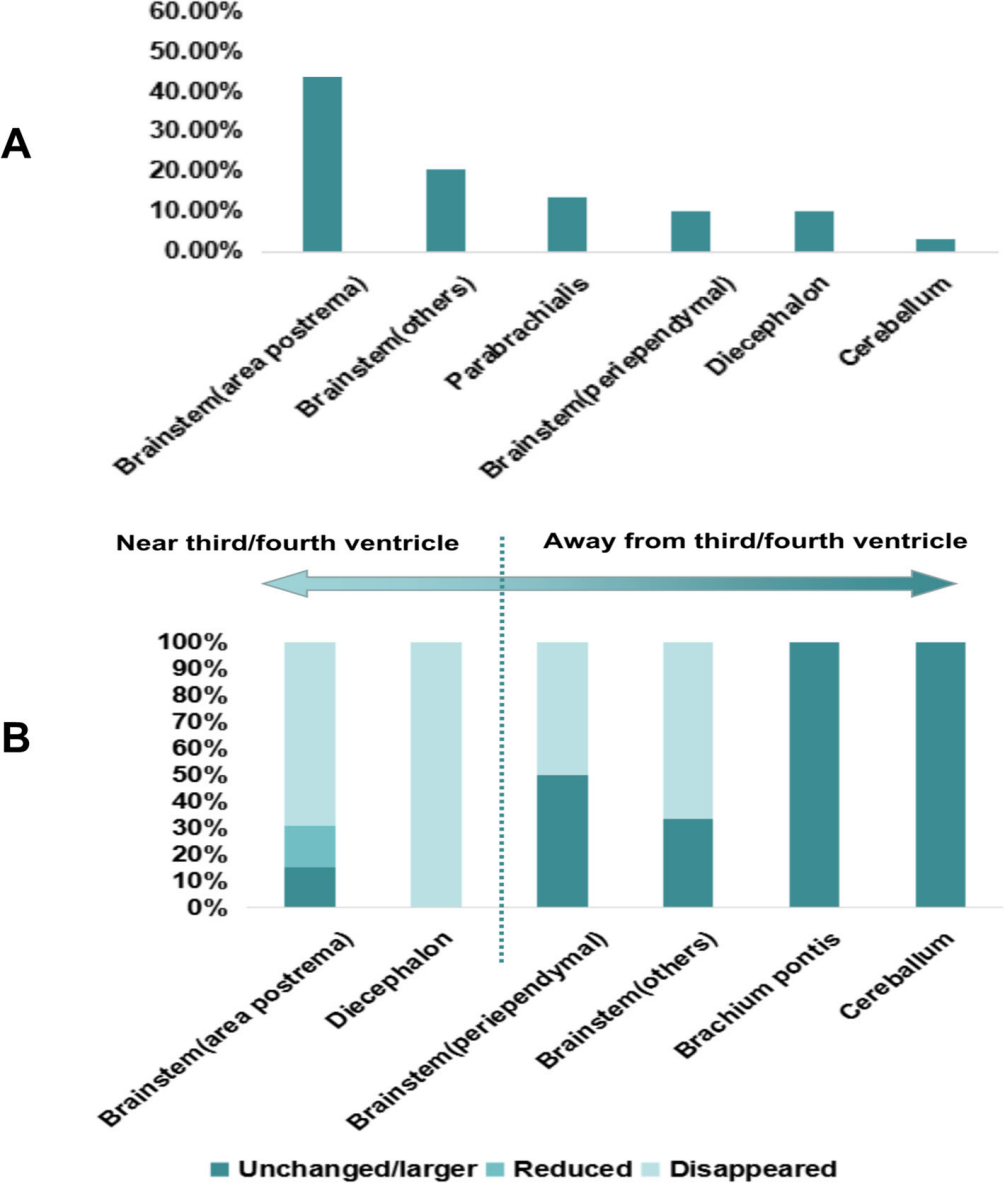
Responsible lesions for non-opticospinal initial symptoms detected in brain MRI and their changes after follow-up in NMOSD-NOSIS. **a**: Responsible lesions for non-opticospinal initial symptoms detected in brain MRI in NMOSD-NOSIS; **b**: Changes of responsible lesions for non-opticospinal initial symptoms in NMOSD-NOSIS after follow-up. NMOSD-NOSIS: NMOSD with non-opticospinal manifestations as initial symptoms

Among these 33 NMOSD-NOSIS patients, 29 underwent brain MRI examinations at the time of onset, 12 had brain MRI examination later during follow-up, and other 2 had no brain MRI examination during the disease course. All of the 12 patients undergoing brain MRI examination later during follow-up had vomiting or hiccups as initial symptoms, and no obvious abnormality was found in their brain MRI. Vomiting or hiccups in 4 patients, headache in 2 and limb spasm in one were not been verified by corresponding MRI-documented lesions at the time of onset. One patient with vomiting or hiccups as initial symptoms had no obvious cerebral lesions on the brain MRI at the time of onset, but developed a lesion in the area postrema after 6 months. Other specified clinical symptoms in those 29 NMOSD-NOSIS patients undergoing brain MRI examination at the time of onset were verified by corresponding MRI-documented lesions. The main responsible lesions for these symptoms were in the area postrema (corresponding to vomiting, hiccup, vertigo, or diplopia; 44.83 %), brainstem (other areas) (corresponding to vomiting, hiccup, vertigo, dsyphagia, or limb weakness/numbness; 20.69 %), parabrachialis (corresponding to vertigo, diplopia, facial paralysis, facial hypoesthesia, or tinnitus; 13.79 %), brainstem (periependymal) (corresponding to hiccup, vertigo, or diplopia; 10.34 %), diencephalon (corresponding to somnolence or psychiatric symptom; 10.34 %), and cerebellum (corresponding to ataxia; 3.45 %) (Fig. 3a). Most of the lesions around the third/fourth ventricle disappeared during follow-up (disappearance rate of lesions: area postrema: 69.23 %; diencephalon: 100 %). The further away from the third/fourth ventricle the lesion occurred, the less likely the lesions were to disappear [The percentage of patients whose lesions disappeared during an average follow-up of 17(IQR:9–46) months in different areas is as follows: periependymal areas in brainstem: 50 %; other areas of the brainstem: 66.67 %; parabrachialis: 0 %; and cerebellum: 0 %] (Fig. 3b).

### Comparison of clinical characteristics, imaging features and long-term clinical outcomes between NMOSD-NOSIS and NMOSD-OSIS patients

**Fig. 4:**
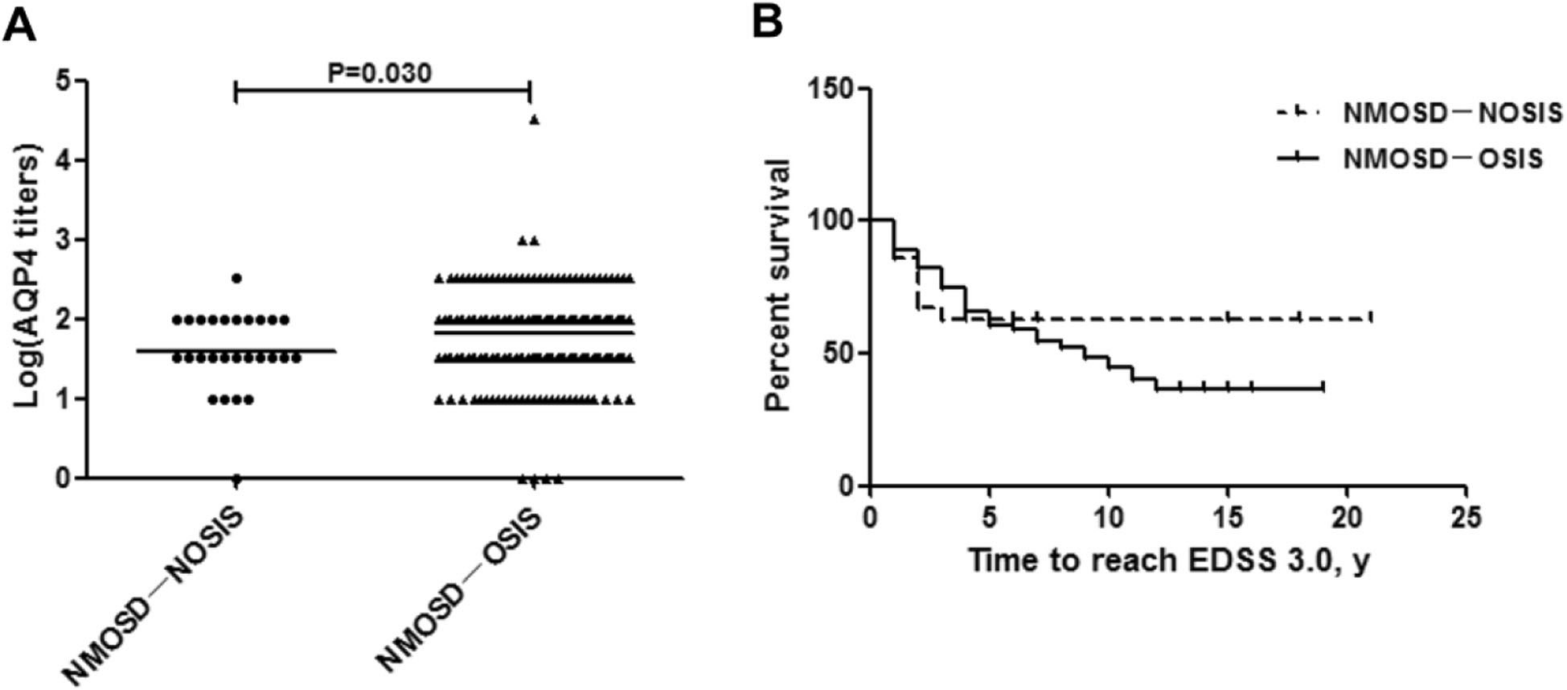
Comparison of serum AQP4 titers and time to reach EDSS 3.0 between NMOSD-NOSIS and NMOSD-OSIS. **a**: Comparison of serum AQP4 titers between NMOSD-NOSIS and NMOSD-OSIS; **b**: Comparison of time to reach EDSS 3.0 between NMOSD-NOSIS and NMOSD-OSIS. NMOSD-NOSIS: NMOSD with non-opticospinal manifestations as initial symptoms; NMOSD-OSIS: NMOSD with opticospinal manifestations as initial symptoms

To prevent the interference of antibody serotypes, we only compared the differences between the NMOSD-NOSIS and NMOSD-OSIS patients who were serum-positive for AQP4 antibodies. As shown in Table 2; Fig. 4, the NMOSD-NOSIS patients had a younger onset age [25(IQR: 20–33) years vs. 36 (IQR: 25–48) years, *P* < 0.001] and lower serum AQP4 titers than the NMOSD-OSIS patients [Log(serum AQP4 titers): 1.59 ± 0.50 vs. 1.81 ± 0.48, *P* = 0.030] (Fig. 4a). The NMOSD-NOSIS patients had lower EDSS scores at onset and follow-up than the NMOSD-OSIS patients [EDSS at onset: 0 (IQR: 0–2.00) vs. 3.00 (IQR: 3.00–4.50), *P* < 0.001; EDSS at follow-up: 2.50 (IQR:1.00–3.00) vs. 3.00 (IQR: 2.00–4.00), *P* = 0.036]. Moreover, the NMOSD-NOSIS patients reached an EDSS of 3.0 later than did the NMOSD-OSIS patients [2.00 (IQR: 1.00–4.00) years vs. 1.00(IQR: 0–4.00) years, *P* = 0.018] (Fig. 4b). After follow-up, there was no difference in the frequency of the ON + MY phenotype between the NMOSD-NOSIS patients and the NMOSD-OSIS patients (57.58 % vs. 61.42 %, *P* = 0.664). When analyzing all 1243 relapse episodes in the 2 groups, we observed that the NMOSD-NOSIS patients had more frequent isolated brain/brainstem attacks during the disease course than did the NMOSD-OSIS patients (12.40 % vs. 3.11 %, *P* < 0.001) (Table 2).

**Table 2:** Comparison about the clinical and imaging features between NMOSD-NOSIS and NMOSD-OSIS patients (serumpositive for AQP4)

	NMOSD-NOSIS *n* = 33	NMOSD-OSIS *n* = 381	*P* value
Age of onset, y, median(IQR)	25.00(20.00–33.00)	36.00(25.00–48.00)	< 0.001
Female,n(%)	31(93.94)	344(90.27)	0.705
ARR, median(IQR)	1.00(0.67–1.31)	0.70(0.46–1.00)	0.133
Coexisting autoimmunity, n(%)	3/33(9.09)	77/381(20.21)	0.121
SLE, n(%)	0(0)	6/381(1.57)	1.000
Sjogren’s syndrome, n(%)	1/33(3.03)	28/381(7.35)	0.564
Hashimoto’s thyroiditis, n(%)	1/33(3.03)	5/381(1.31)	0.974
Hyperthyroidism or subclinical hyperthyroidism, n(%)	1/33(3.03)	15/381(3.94)	1.000
EDSS at onset, median(IQR)	0(0–2.00)	3.00(3.00–4.50)	< 0.001
EDSS at the last follow-up, median(IQR)	2.50(1.00–3.00)	3.00(2.00–4.00)	0.036
Clinical phenotype			
ON, n(%)	0/330(0)	43/381(11.29)	0.082
MY, n(%)	0/33(0)	69/381(18.11)	0.007
ON + MY, n(%)	19/33(57.58)	234/381(61.42)	0.664
Brain/brainstem, n(%)	4/33(12.12)	0/381(0)	< 0.001
Others, n(%)	10/33(30.30)	35/381(9.19)	0.001
Relapse phenotype			
ON, n(%)	26/129(20.16)	333/1114(29.89)	0.021
MY, n(%)	55/129(42.64)	496/1114(44.52)	0.683
ON + MY, n(%)	15/129(11.63)	215/1114(19.30)	0.034
Brain/brainstem, n(%)	16/129(12.40)	38/1114(3.11)	< 0.001
Others, n(%)	17/129(13.18)	32/1114(2.87)	< 0.001
Brain MRI			
Abnormal throughout the course, n(%)	22/31(70.97)	168/246(68.29)	0.762
Cerebral hemisphere, n(%)	12/31(38.71)	120/246(48.78)	0.290
Diecephalon, n(%)	3/31(9.68)	26/246(10.57)	1.000
Cerebellum, n(%)	4/31(12.90)	14/246(5.69)	0.251
Brainstem(area postrema), n(%)	13/31(41.94)	47/246(19.11)	0.004
Brainstem(periepiendymal), n(%)	5/31(16.13)	11/246(4.47)	0.027
Brainstem(others), n(%)	16/31(51.61)	82/246(33.33)	0.045
Optic nerve MRI			
Abnormal, n(%)	5/7(71.43)	94/113(83.19)	0.778
> 1/2 optic nerve, n(%)	2/7(28.57)	48/113(42.48)	0.742
Spinal MRI			
Abnormal throughout the course, n(%)	24/31(77.42)	292/345(84.67)	0.293
LETM, n(%)	17/31(54.84)	253/345(73.33)	0.028
Cervical segments, n(%)	14/31(45.16)	84/345(24.35)	0.011
Thoracic segments, n(%)	1/31(3.23)	62/345(17.97)	0.035
Cervical + thoracic segments, n(%)	9/31(29.03)	147/345(42.61)	0.142
Follow-up,y,median(IQR)	4.00(3.00–8.00)	5.00(3.00–9.00)	0.946

*NMOSD* neuromyelitis optica spectrum disorders, *NMOSD-NOSIS* NMOSD with non-opticospinal manifestations as initial symptoms, *NMOSD-OSIS* NMOSD with opticospinal manifestations as initial symptoms, *ARR *annual relapse rate, *IQR* Interquartile range, *SLE* Systemic lupus erythematosus, *ON *optic neurotis, *MY *myelitis, *CSF *cerebral spinal fluid, *EDSS *expanded disability status scale, *MRI *magnetic resonance imaging, *LETM *longitudinal extensive transverse myelitis

Of the total 414 patients, 18 patients did not have brain or spinal cord MRI data. Three hundred and six patients had baseline brain or spinal cord MRI data, and 163 patients underwent a brain or spinal cord MRI reexamination during the follow-up. All lesions detected in baseline and follow-up MRI examinations were recorded and included in the analysis. Brainstem lesions were more commonly present in NMOSD-NOSIS patients than in NMOSD-OSIS patients [Brainstem (area postrema): 41.94 % vs. 19.11 %, *P* = 0.004; Brainstem (periependymal): 16.13 % vs. 4.47 %, *P* = 0.027; Brainstem (others): 51.61 % vs. 33.33 %, *P* = 0.045]. NMOSD-NOSIS patients had less frequent longitudinally extensive transverse myelitis (LETM), were more likely to have cervical spinal cord lesions, and were less likely to have thoracic spinal cord lesions than were NMOSD-OSIS patients (LETM: 54.84 % vs. 73.33 %, *P* = 0.028; cervical segment: 45.16 % vs. 24.35 %, *P* = 0.011; thoracic segment: 3.23 % vs. 17.97 %, *P* = 0.035) (Table 2).

Evaluation of the pre- and post-treatment ARRs for the main treatment modalities between NMOSD-NOSIS and NMOSD-OSIS patients is shown in Table 3. Three hundred and three out of 414 patients treated with immunosuppressive agents for more than 1 year were included in the analysis; 178 were administered azathioprine (2 mg/kg daily), 105 were given mycophenolate mofetil (1 g/day), and 20 were given rituximab [500 mg through intravenous infusion, repeated 2 weeks later. This cycle was repeated every 6 months (4 cycles in total for each patient)]. ARR decreased significantly after the three major immunosuppressant therapies in both the NMOSD-OSIS and NMOSD-NOSIS groups.

**Table 3:** Evaluation of pre- and post-treatment ARRs in NMOSD-NOSIS and NMOSD-OSIS (serumpositive for AQP4)

Treatment	Therapy duration,y(range)	Patient groups(n)	Pre-treatment	Post-treatment	*P* value	Relapse-free within 1 year, n(%)	Relapse-free within 2 year, n(%)
Azathioprine	2.00(1.50–3.00)	NMOSD-NOSIS *n* = 17	2.00(0.50- 3.00)	0.50(0–1.00)	0.003	11/17(64.71)	7/16(43.75)
	3.00(2.00–5.00)	NMOSD-OSIS *n* = 161	1.00(0.50–1.53)	0.27(0–0.67)	< 0.001	103/160(64.38)	70/138(50.72)
Mycophenolate mofetil	2.00(2.00–4.00)	NMOSD-NOSIS *n* = 10	2.00(1.45–2.25)	0.25(0–0.56)	0.008	8/10(80.00)	4/9(44.44)
	2.00(1.00–3.00)	NMOSD-OSIS *n* = 95	0.80(0.50–1.25)	0(0–0.50)	< 0.001	77/95(81.05)	38/75(50.67)
Rituximab	1.00(1.00–1.00)	NMOSD-NOSIS *n* = 3	1.25(--)	0(--)	0.285	2/3(66.67)	--
	2.00(1.00–2.50)	NMOSD-OSIS *n* = 17	1.00(0.71- 2.00)	0.33(0–1.25)	0.055	10/17(58.82)	6/13(46.15)

*NMOSD* neuromyelitis optica spectrum disorders, *NMOSD-NOSIS* NMOSD with non-opticospinal manifestations as initial symptoms, *NMOSD-OSIS* NMOSD with opticospinal manifestations as initial symptoms, *ARR *annual relapse rate. P:comparison of pre- and post-treatment ARR

## Discussion

Since the clinical characteristics and long-term outcomes of patients with NMOSD-NOSIS remain unknown, we retrospectively evaluated such patients in comparison with NMOSD-OSIS patients. Whereas it was not clear whether all patients with NMOSD-NOSIS will eventually develop ON/MY, our research showed that the majority do (88.37 %). Only 11.63 % of all patients with NMOSD-NOSIS had non-opticospinal manifestations throughout the disease course. However, the follow-up period of NMOSD-NOSIS patients who showed restricted non-opticospinal manifestations was shorter than that of NMOSD-NOSIS patients who did not develop ON/MY; thus, it is possible that these NMOSD-NOSIS patients with restricted non-opticospinal manifestations will develop ON/MY before subsequent follow-ups.

NMOSD-NOSIS is easily misdiagnosed as Wernicke encephalopathy (peri-third ventricle region involvement), GBS (medulla oblongata involvement), or digestive system diseases (area postrema involvement). As NMOSD-NOSIS patients have a high probability of developing ON/MY, it is important to recognize that NMOSD can initially present with non-opticospinal symptoms. Our study revealed that all the non-opticospinal manifestations in NMOSD were brain/brainstem symptoms. Most of the non-opticospinal manifestations were area postrema syndrome (vomiting and hiccups), acute brainstem syndrome (vertigo, diplopia), and acute diencephalic syndrome (somnolence), which are now recognized as being among the core clinical characteristics of NMOSD, in addition to ON and LETM, and have been included in the newly proposed diagnostic criteria [5, 7]. More importantly, we identified other non-opticospinal manifestations, such as ataxia, facial paralysis, facial hypoesthesia, headache, psychiatric symptoms, limb spasms, and tinnitus; and the causative lesions were located in the cerebellum, parabrachialis, thalamus, and other nonspecific parts of the brainstem. These symptoms and their responsible lesions expand our understanding of the spectrum of initial symptoms of NMOSD, which may facilitate the early recognition of NMOSD-NOSIS patients.

Both non-opticospinal symptoms and associated medullary MRI lesions, particularly in the peri-third/fourth ventricle areas (diencephalon and area postrema), were reversible in many NMOSD-NOSIS patients. Compared with opticospinal symptoms, non-opticospinal symptoms recovered better. The CR rate of non-opticospinal symptoms was higher than that of opticospinal symptoms. Most of the lesions near the third and fourth ventricles (area postrema and diencephalon) resolved during follow-up. In addition, 12 patients presented with vomiting or hiccups as initial symptoms (suggesting area postrema involvement, but brain MRI was not performed at the time of onset). During the follow-up, no area postrema lesions were found on their brain MRIs, suggesting that the area postrema lesions may have disappeared.

It is worth noting that five NMOSD-NOSIS patients had a normal appearance on brain MRI when they complained of hiccups or vomiting. It is conceivable that the early the brain imaging may have missed evolving lesions. This postulation is supported by the observation that medullary lesions were detected in a subsequent MRI in one patient who had initially presented with area postrema syndrome 6 months prior. Another possible explanation is that the lesions were too small to be detected by MRI. Therefore, if a patient complains of nausea, vomiting, or other brainstem symptoms, which cannot be explained clinically and with normal-appearing MR images, the possibility of NMOSD should always be considered, and the serum AQP4 antibody should be assessed.

The clinical characteristics of NMOSD-NOSIS patients differed from those of NMOSD-OSIS patients. NMOSD-NOSIS patients had a younger onset age and lower serum AQP4 titers than NMOSD-OSIS patients. Although NMOSD-NOSIS patients may develop ON/MY during follow-up, the frequency of non-ON/MY relapse episodes remained higher than that in NMOSD-OSIS patients. NMOSD-NOSIS patients more commonly had brainstem lesions, more cervical and less thoracic involvement, and less frequent LETM, than NMOSD-OSIS patients.

NMOSD-NOSIS patients had better long-term clinical outcomes. After a similar follow-up period, NMOSD-NOSIS patients had lower EDSS scores and took longer to reach EDSS scores of 3.0 than NMOSD-OSIS patients. This may be due to the following reasons: (1) The brain lesions in NMOSD-NOSIS patients were mostly concentrated in the peri-third/fourth ventricle (diencephalon and area postrema) or periependymal areas in the brainstem, avoiding the pyramidal tract, and thus had little effect on motor function. (2) NMOSD-NOSIS patients had a younger age of onset than NMOSD-OSIS patients. Several previous studies have reported poor clinical outcomes in older-onset patients with NMOSD [10, 11]. (3) The immunopathogenesis of NMO lesions in the peri-third/fourth ventricle areas may be different from the typically destructive NMO lesions that predominate in the optic nerves or spinal cord. Immunohistochemical analyses of archival brain, spinal cord, and optic nerve tissues obtained from patients with NMO have demonstrated a novel NMO lesion phenotype in the medullary floor of the fourth ventricle (including the area postrema), which exhibits loss of AQP4 and contains inflammatory cells, but lacks demyelination or necrosis [12, 13]. Binding of NMO-IgG to AQP4 in the peri-third/fourth ventricle areas may be less efficient at activating the complement system [11]. The peri-third/fourth ventricle areas in the brainstem are close to the cerebrospinal fluid (CSF) circulation system, and these circumventricular neural structures are devoid of a blood-brain barrier (BBB) [14, 15]. This anatomical site could thus serve as an entry point for circulating AQP4-IgG entry into the CSF. We hypothesized that the circulating AQP4 antibody entered the CSF circulation through the weak BBB in the peri-third/fourth ventricle areas, thus reducing the concentration of AQP4-IgG in the serum and nerve tissue, and relieving the inflammatory response and neurological damage in these areas. The finding that the serum anti-AQP4 titers in NMOSD-NOSIS patients were lower than those in NMOSD-OSIS patients in our study supports our point of view. This hypothesis needs further corroboration with evaluation of the CSF AQP4-IgG titers in both NMOSD-NOSIS and NMOSD-OSIS patients in the future.

Our results should be interpreted with caution in light of several limitations. One is our relatively small sample size of NMOSD-NOSIS cases. Another is that we used retrospective data from a single center, thus the potential selection bias and missing data may affect the interpretations of the findings and the conclusions of the study. Despite these limitations, the long-term follow-up of these cases revealed the clinical characteristics and outcomes of NMOSD-NOSIS patients to a large extent. A larger multi-center prospective study with CSF AQP4-IgG data is needed in the future.

## Conclusions

Patients with NMOSD with NOSIS have a younger age of onset, lower serum AQP4 antibody titers, and better clinical outcomes than do patients with OSIS.

## Data Availability

The datasets used and/or analyzed during the current study are available from the corresponding author on reasonable request.

## Abbreviations

ARR: Annual relapse rate
AQP4: Anti-aquaporin-4
BBB: Blood-brain barrier
CNS: Central nervous system
CSF: Cerebrospinal fluid
CR: Complete remission
EDSS: Expanded Disability Status Scale
GBS: Guillain-Barre Syndrome
IgG: Immunoglobulin G
IQR: Interquartile range
LETM: Longitudinally extensive transverse myelitis
MR: Magnetic resonance
MRI: Magnetic resonance imaging
MY: Myelitis
NMO: Neuromyelitis optica
NMOSD: Neuromyelitis optica spectrum disorders
NOSIS: Non-opticospinal manifestations as initial symptoms
ON: Optic neuritis
TE: Echo time
TR: Repetition time

## References

[1] Devic E. Myélite aiguë dorso-lombaire avec névrite optique, Autopsie. Congrès français de médecine. 1895:434–9.

[2] Gault F. De la neuromyélite optique aiguë. 1894.

[3] Lennon VA, Wingerchuk DM, Kryzer TJ, Pittock SJ, Lucchinetti CF, Fujihara K (2004). A serum autoantibody marker of neuromyelitis optica: distinction from multiple sclerosis. Lancet.

[4] Wingerchuk DM, Lennon VA, Pittock SJ, Lucchinetti CF, Weinshenker BG (2006). Revised diagnostic criteria for neuromyelitis optica. Neurology.

[5] Wingerchuk DM, Banwell B, Bennett JL, Cabre P, Carroll W, Chitnis T (2014). Revised diagnostic criteria for neuromyelitis optica spectrum disorders. Neurology.

[6] Lana-Peixoto MA, Talim N. Neuromyelitis Optica Spectrum Disorder and Anti-MOG Syndromes. Biomedicines. 2019;7 2; doi:10.3390/biomedicines7020042. 10.3390/biomedicines7020042 PMC6631227 31212763

[7] Wingerchuk DM, Banwell B, Bennett JL, Cabre P, Carroll W, Chitnis T (2015). International consensus diagnostic criteria for neuromyelitis optica spectrum disorders. Neurology.

[8] Filippi M, Rocca MA, Ciccarelli O, De Stefano N, Evangelou N, Kappos L (2016). MRI criteria for the diagnosis of multiple sclerosis: MAGNIMS consensus guidelines. Lancet Neurol.

[9] Kleiter I, Gahlen A, Borisow N, Fischer K, Wernecke KD, Wegner B (2016). Neuromyelitis optica: Evaluation of 871 attacks and 1,153 treatment courses. Ann Neurol.

[10] Kitley J, Leite MI, Nakashima I, Waters P, McNeillis B, Brown R, et al. Prognostic factors and disease course in aquaporin-4 antibody-positive patients with neuromyelitis optica spectrum disorder from the United Kingdom and Japan. Brain. 2012;135(Pt 6):1834–49. doi:10.1093/brain/aws109. 10.1093/brain/aws109 22577216

[11] Mao Z, Yin J, Zhong X, Zhao Z, Qiu W, Lu Z (2015). Late-onset neuromyelitis optica spectrum disorder in AQP4-seropositivepatients in a Chinese population. BMC Neurol.

[12] Roemer SF, Parisi JE, Lennon VA, Benarroch EE, Lassmann H, Bruck W (2007). Pattern-specific loss of aquaporin-4 immunoreactivity distinguishes neuromyelitis optica from multiple sclerosis. Brain.

[13] Apiwattanakul M, Popescu BF, Matiello M, Weinshenker BG, Lucchinetti CF, Lennon VA (2010). Intractable vomiting as the initial presentation of neuromyelitis optica. Ann Neurol.

[14] Duvernoy HM, Risold PY (2007). The circumventricular organs: an atlas of comparative anatomy and vascularization. Brain Res Rev.

[15] Bentivoglio M, Kristensson K, Rottenberg ME (2018). Circumventricular Organs and Parasite Neurotropism: Neglected Gates to the Brain?. Front Immunol.

